# RNase L promotes the formation of unique ribonucleoprotein granules distinct from stress granules

**DOI:** 10.1074/jbc.RA119.011638

**Published:** 2020-01-02

**Authors:** James M. Burke, Evan T. Lester, Devin Tauber, Roy Parker

**Affiliations:** ‡ Department of Biochemistry, University of Colorado, Boulder, Colorado 80303; § Howard Hughes Medical Institute, University of Colorado, Boulder, Colorado 80303

**Keywords:** stress granule, processing body (P-body), RNA turnover, RNA degradation, double-stranded RNA (dsRNA), innate immunity, lipid droplet, Protein kinase R (PKR), Ribonuclease L (RNase L)

## Abstract

Stress granules (SGs) are ribonucleoprotein (RNP) assemblies that form in eukaryotic cells as a result of limited translation in response to stress. SGs form during viral infection and are thought to promote the antiviral response because many viruses encode inhibitors of SG assembly. However, the antiviral endoribonuclease RNase L also alters SG formation, whereby only small punctate SG-like bodies that we term RNase L–dependent bodies (RLBs) form during RNase L activation. How RLBs relate to SGs and their mode of biogenesis is unknown. Herein, using immunofluorescence, live-cell imaging, and MS-based analyses, we demonstrate that RLBs represent a unique RNP granule with a protein and RNA composition distinct from that of SGs in response to dsRNA lipofection in human cells. We found that RLBs are also generated independently of SGs and the canonical dsRNA-induced SG biogenesis pathway, because RLBs did not require protein kinase R, phosphorylation of eukaryotic translation initiation factor 2 subunit 1 (eIF2α), the SG assembly G3BP paralogs, or release of mRNAs from ribosomes via translation elongation. Unlike the transient interactions between SGs and P-bodies, RLBs and P-bodies extensively and stably interacted. However, despite both RLBs and P-bodies exhibiting liquid-like properties, they remained distinct condensates. Taken together, these observations reveal that RNase L promotes the formation of a unique RNP complex that may have roles during the RNase L–mediated antiviral response.

## Introduction

Eukaryotic cells limit translation initiation in response to numerous and diverse stresses, including oxidative stress, endoplasmic reticulum stress, misfolded proteins, metabolic imbalance, heat shock, and viral infection. In response to these stresses, one or more eIF2α kinases (PERK, GCN2, PKR, HRI) phosphorylate eIF2α on serine 51 (p-eIF2α), which inhibits the guanine nucleotide exchange activity of eIF2B (1). This results in inhibition of translation initiation and an accumulation of cytoplasmic nontranslating mRNAs, which leads to the generation of stress granules (SGs)^2^ (2).

SGs are conserved RNA–protein complexes that contain nontranslating mRNAs and numerous RNA-binding proteins, such as G3BP1, PABPC1, TIA1, FMRP, and Ataxin-2 (3). Several functions for stress granules have been proposed and include cell survival, cellular signaling, and regulation of mRNA partitioning and turnover (4). In addition, SGs and their associated RBPs are implicated in promoting the formation of pathogenic RNP aggregates observed in several neurodegenerative diseases, such as amyotrophic lateral sclerosis, Alzheimer's disease, and frontotemporal dementia (5–12). Therefore, much interest has been garnered in understanding the biogenesis and functions of SGs during diverse stresses, which remain incompletely understood.

SGs and SG-like assemblies are also generated in response to viral infection (reviewed in Ref. 13). Several key antiviral pattern recognition receptors and antiviral proteins have been reported to localize to SGs, including OAS, RNase L, PKR, MDA-5, and RIG-I (14–16). Furthermore, many viruses inhibit SG assembly. Combined, these observations suggest that SGs may serve as antiviral signaling hubs and sequester host and/or viral mRNAs/proteins to reduce viral replication. However, to promote translation of antiviral mRNAs sequestered into SGs, a model has been proposed where periodic reinitiation of canonical translation and disassembly of SGs is mediated by induction of GADD34, which is preferentially translated during inhibition of canonical translation, is transcriptionally induced by RLR signaling, and promotes the dephosphorylation of p-eIF2α (17). Nevertheless, the biogenesis pathways and functions of SGs during the host response to dsRNA and viral infection remain enigmatic.

Recently, we and others demonstrated that the antiviral RNase L endonuclease promotes widespread turnover of cellular mRNAs in response to foreign dsRNA, limiting global translation activity (18, 19). Concurrently, RNase L promotes the translocation of PABPC to the nucleus, limits canonical SG assembly, and promotes the assembly of small punctate RNase L–dependent bodies (RLBs). In this study, we examine the relationship between canonical SGs and RLBs. We show that dsRNA-induced RLBs and SGs have different biogenesis pathways, RNA and protein composition, and biophysical characteristics. Thus, activation of RNase L triggers the formation of unique RNP granules, which may influence the process of viral infection and/or the innate immune response.

## Results

### RLBs are distinct from P-bodies, which are unaffected by dsRNA and RNase L responses

**Figure 1.**
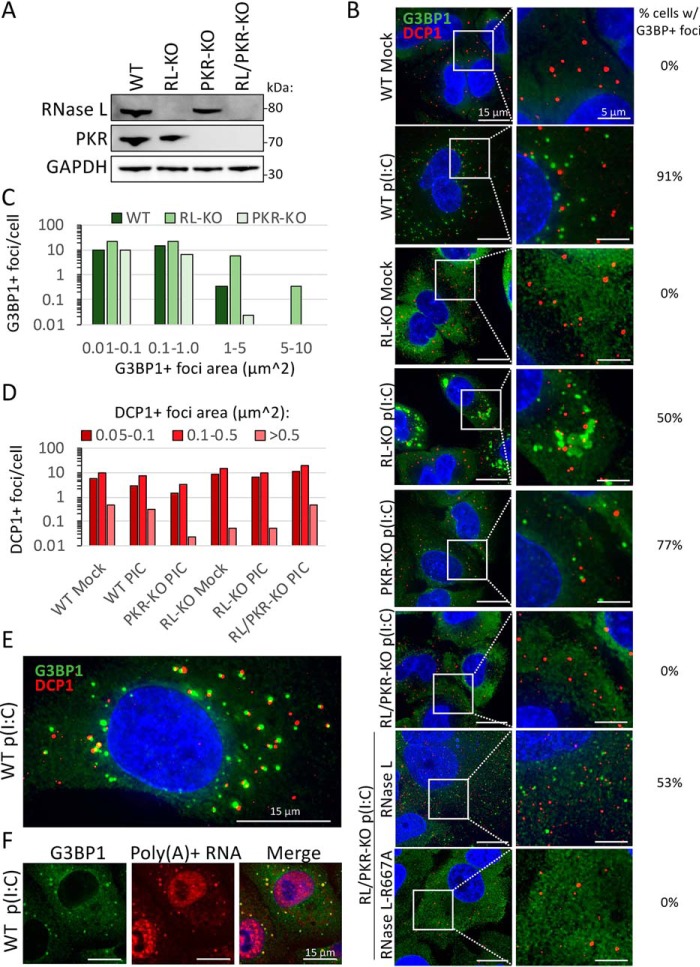
**RNase L inhibits SG but not PB assembly and promotes RLB assembly.** *A*, Western blotting analysis of PKR and RNase L in parental (WT), RNase L–KO (RL-KO), PKR-KO, and RNase L/PKR double knockout (RL/PKR-KO) A549 cells. *B*, IF for G3BP1 (SG marker) and DCP1b (PB marker) in the indicated A549 cell lines transfected with or without poly(I·C). To calculate the percentage of cells with G3BP1+ foci (RLBs or SGs), 25–195 cells from between 3 and 15 fields of view were analyzed. *C*, histogram of the number of G3BP1-positive foci binned by area as represented in *B. D*, histogram of the number of DCP1b-positive foci binned by area as represented in *B. E*, similar to *B* but depicting RLB and PB association. *F*, IF for G3BP1 and FISH for poly(A)+ RNA in WT cells 4 h post-poly(I·C).

We previously reported that RNase L catalytic activity inhibits the assembly of canonical SGs and promotes the assembly of small punctate G3BP1-positive foci termed RNase L–dependent bodies (18). RLBs are similar to the size and morphology of P-bodies, cytoplasmic mRNP complexes that can associate with SGs and contain mRNA turnover machinery (20, 21). This led to the hypothesis that RLBs are hybrids of P-bodies and SGs, whereby G3BP1 enters P-bodies because of RNase L–mediated mRNA decay. To determine whether RLBs are distinct from P-bodies and/or whether RNase L affects P-body assembly, we performed immunofluorescence (IF) for G3BP1 (SG and RLB marker) and DCP1 (P-body marker) in WT, RL-KO, PKR-KO, and RL/PKR-KO A549 cells following poly(I·C) transfection (Fig. 1, *A* and *B*).

Consistent with our previous findings, only RLBs assembled in WT and PKR-KO cells post-poly(I·C), whereas canonical SGs assembled in RL-KO cells but not in RL/PKR-KO A549 cells (Fig. 1, *B* and *C*). The catalytic activity of RNase L is required for RLB assembly in response to poly(I·C) lipofection, because stable expression of RNase L but not catalytically inactive RNase L–R667A rescued RLB formation in RL/PKR-KO cells (Fig. 1*B*) (18). Thus, RNase L activation generates RLBs and inhibits PKR-dependent SGs.

Importantly, P-bodies were observed as distinct complexes from RLBs (Fig. 1*B*). Interestingly, the number and size of P-bodies did not notably change in response to poly(I·C) and were similar in WT, RL-KO, and PKR-KO cells (Fig. 1, *B* and *D*). These observations reveal that P-body morphology is unaffected by RNase L–mediated RNA decay. Moreover, these observations reveal that dsRNA lipofection and PKR-induced translational repression do not affect P-body morphology. These data demonstrate that RLBs are unique assemblies distinct from P-bodies.

### P-bodies and RLBs stably interact

Analysis of RLBs and P-bodies revealed extensive docking between the two granules (Fig. 1*E*). This docking is similar to the docking of SGs with P-bodies in RL-KO cells (Fig. 1*B*), which has been previously observed (22). Because RLBs contain RNA, as assessed by poly(A)+ FISH (Fig. 1*F*) (18), this provides another example of docking between RNP granules in cells.

Strikingly, live-cell imaging of U-2 OS cells stably expressing mRFP-DCP1 and GFP–G3BP1 post-poly(I·C) revealed that P-bodies and RLBs can stably associate for long periods of time (>30 min) (Movie S1). This is in contrast to SGs, which only transiently associate with P-bodies (Movie S2) (22). Moreover, we observed that RLBs and P-bodies closely associate, whereby fluorescence between the two commonly overlapped (Fig. 1*E* and Movie S1). In contrast, SGs and P-bodies generally remained separated (Fig. 1*B* and Movie S2).

We observed several instances in which multiple RLBs associated with a single P-body merge with one another. Likewise, we observed multiple P-bodies associated with an RLB merge with one another (Movie S3). However, we did not observe merging between P-bodies and RLBs despite their stable interactions (Movies S1 and S3). The spherical nature of RLBs and P-bodies, as well as the ability of RLBs and P-bodies to fuse in a homotypic manner, indicate that RLBs and P-bodies are separate and distinct liquid-like condensates with the ability to stably associate but not fuse with one another.

### RLBs and SG have distinct protein composition

**Figure 2.**
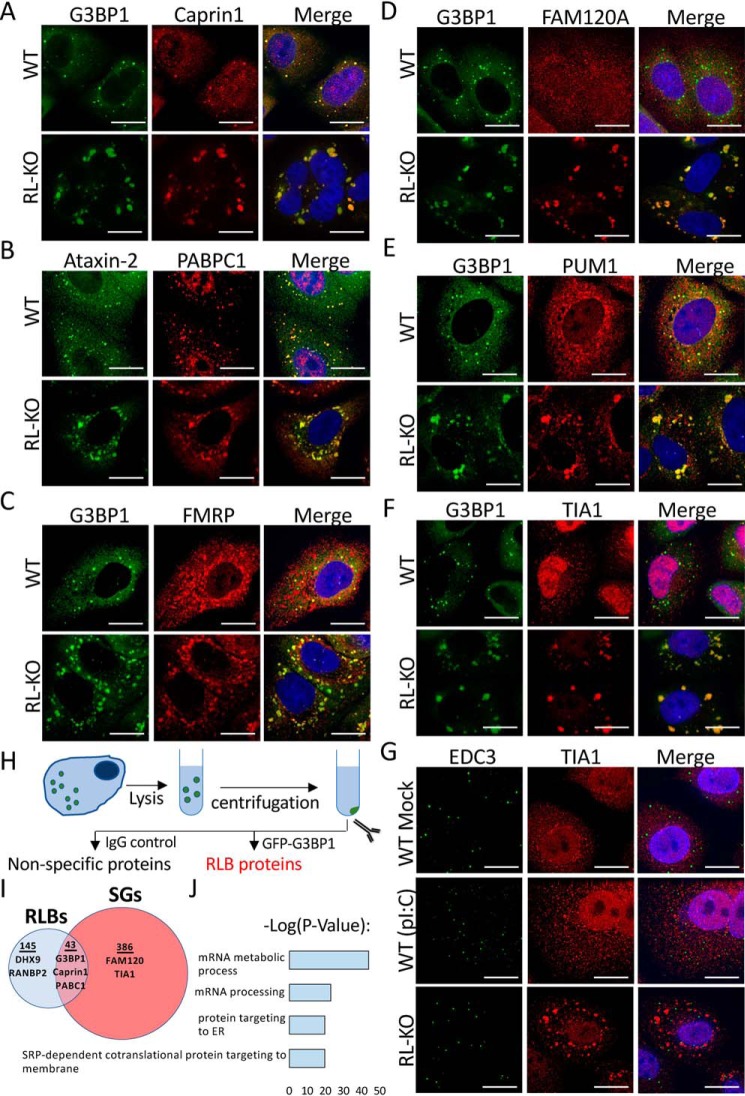
**RNase L regulates the localization of RBPs to mRNP complexes during dsRNA stress.** *A–G*, IF for indicated RBPs in WT and RL-KO A549 cells post-poly(I·C) treatment. *Scale bars* represent 15 μm. EDC3 in *G* is a P-body marker. *H*, schematic of protocol to enrich for RLB-associated proteins. *I*, Venn diagram of proteins associated with RLBs isolated from U-2 OS GFP–G3BP1 cells and proteins identified in SGs from Refs. 3, 40, and 41. *J*, Gene Ontology analysis of RLB-associated proteins identified by MS.

To determine how RLBs are related to SGs, we examined their protein composition via immunofluorescence assays for common SG-associated RBPs. We observed that RLBs contain multiple SG-associated proteins (G3BP1, PABPC1, Caprin1, and Ataxin-2) (Fig. 2, *A* and *B*). However, several RBPs enriched in dsRNA-induced SGs are not as enriched in RLBs (FMRP, FAM120A, PUM1, and TIA1) (Fig. 2, *C–F*). We note that TIA1 formed small punctate foci distinct from both RLBs and P-bodies in WT cells (Fig. 2, *F* and *G*), suggesting that TIA1 is redistributed to a separate mRNP complex in response to RNase L activation. Thus, RLBs contain some RBPs commonly enriched in SGs, although partitioning of these RBPs is differential, with several SG-associated RBPs having reduced partitioning to RLBs.

We next wanted to comprehensively assess the composition of RLBs. To do this, we induced RLBs in U-2 OS GFP–G3BP1 cells via poly(I·C) lipofection. We then employed a strategy previously used to identify SG-associated proteins (3), whereby we enriched for RLBs via differential centrifugation, immunoprecipitated GFP–G3BP1, and performed MS on GFP–G3BP1–associated proteins (Fig. 2*H*). We identified 188 proteins associated with RLBs that only partially overlap with the SG proteome (Fig. 2*I* and Data File S1), which was largely consistent with our IF analysis. Gene Ontology analysis of RLB-associated proteins revealed that RLBs are enriched in proteins involved in mRNA metabolism and processing, protein targeting to the endoplasmic reticulum, and SRP-dependent co-translational protein targeting to membrane (Fig. 2*J*). These data indicate that RLBs have a unique proteome that defines them as an RNase L–induced RNP granule distinct from SGs and may function in mRNA processing and/or regulation of translation.

### RNase L activation inhibits canonical SG assembly and promotes SG disassembly

**Figure 3.**
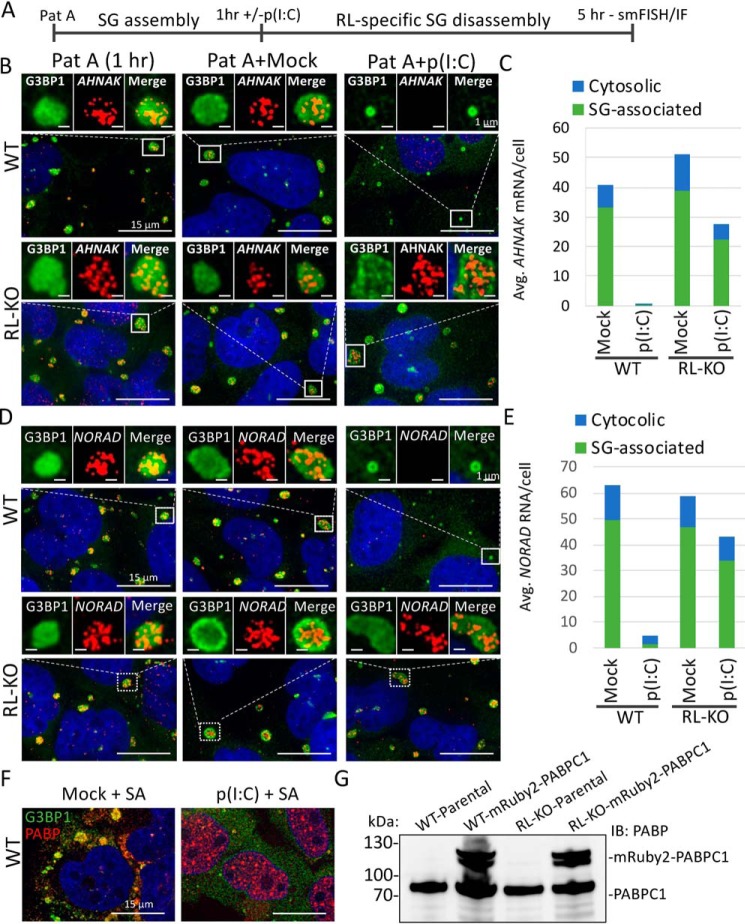
**RNase L inhibits the assembly and promotes the disassembly of SGs.** *A*, schematic representing the experiment in *B–E*. WT or RL-KO U-2 OS cells were treated with 100 nm of pateamine A (*Pat A*) for 1 h. The cells were then either mock-transfected or transfected with poly(I·C). 4 h later IF/smFISH was performed. *B*, IF for G3BP1 and smFISH for *AHNAK* mRNA. *C*, quantification of *AHNAK* mRNA in the cytosol and associated with G3BP1-positive foci as represented in *B. D*, IF for G3BP1 and smFISH for *NORAD* lncRNA. *E*, quantification of *NORAD* lncRNA in the cytosol and associated with G3BP1-positive foci as represented in *D. F*, IF for SG markers PABPC1 and G3BP1 in A549 cells that were transfected with or without poly(I·C). 4 h later, the cells were treated with 500 μm sodium arsenite (*SA*). *G*, immunoblot analysis of PABPC1 in WT and RL-KO cells transduced with lentivirus encoding mRuby-2–PABPC1.

In principle, RLBs could form *de novo* or could form from RNase L degrading mRNAs within SGs, thereby altering their composition to form RLBs. To test whether RNase L can promote the disassembly of SGs and/or convert SGs into RLBs, we first treated WT or RL-KO cells with pateamine A, which inhibits eIF4A and leads to the formation of stable SGs (Fig. 3*A*) (23). We then transfected cells with or without poly(I·C) and performed smFISH for SG-enriched RNAs (24), *AHNAK* mRNA and *NORAD* lncRNA, and IF for G3BP1.

We observed that preformed pateamine A–induced SGs were disassembled in an RNase L–dependent manner concurrent with the loss of SG-enriched *AHNAK* mRNA and NORAD lncRNA (Fig. 3, *B–E*). Live-cell imaging of this process revealed a rapid reduction in pateamine A–induced SG size post-poly(I·C) in WT but not RL-KO cells (Movies S4 and S5). Interestingly, SGs were not completely disassembled but instead reduced in size comparable with that of RLBs, suggesting that RLBs can be generated via RL-mediated SG disassembly. These data argue that RNase L can disassemble SGs via degradation of SG-associated mRNAs, leading to RLB assemblies.

To test whether RNase L can inhibit SG assembly, we first transfected WT cells with or without poly(I·C) and after 4 h subsequently treated the cells with sodium arsenite. We observed that RNase L activation via poly(I·C) transfection inhibited the assembly of large sodium arsenite–induced SGs and instead led to the formation of small G3BP1 and PABC1 containing foci, which we interpret as RLBs (Fig. 3*F*). Thus, prior activation of RNase L blocks the formation of canonical SGs.

### dsRNA-induced RLBs form independently of SGs

Because RNase L disassembled pateamine A–induced SGs into RLB-sized assemblies, we next addressed whether RLBs form via SG disassembly or whether RLBs typically form independently of SGs. To do this, we generated WT and RL-KO A549 cells that stably express mRuby-2–PABPC1 via lentiviral transduction and performed live-cell imaging post-poly(I·C) (Fig. 3*G*).

WT cells only formed RLBs post-poly(I·C) (Movie S6 and S7). This indicates that PKR activation rarely occurs without and does not precede RNase L co-activation in response to poly(I·C) transfection in A549 cells. Moreover, RLBs form in a greater percentage of WT cells (87%) compared with SGs that form in RL-KO cells (44%), and RLB assembly (261 min on average) is faster than SG assembly (400 min on average) (Movies S6 and S8). These observations indicate that RLBs do not typically originate from RNase L–mediated disassembly of preformed SGs but instead are assembled via a distinct pathway independently of SGs.

### RLBs have a distinct biogenesis pathway compared with SGs

The ability of RLBs to assemble independently of PKR, and their requirement for RNase L is in contrast to SGs, which require PKR for their assembly but are inhibited by RNase L (Fig. 1) (18). These observations, combined with the increased frequency and faster biogenesis rate of RLBs, suggest that dsRNA-induced RLBs and SGs assemble via distinct biogenesis pathways. To address this, we first examined the dependence of RLBs and SGs on phosphorylation of eIF2α by transfecting either WT mouse embryonic fibroblasts (MEF-WT) or MEFs with a knockin eIF2α S51A mutation (MEF–eIF2α–S51A) with poly(I·C).

**Figure 4.**
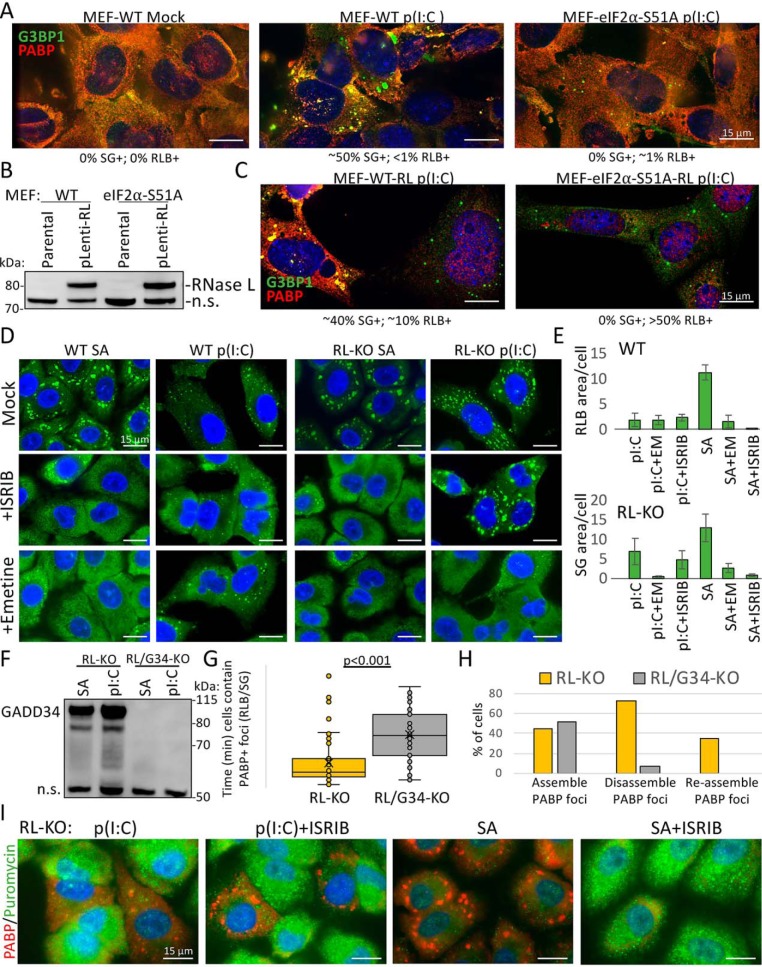
**RLBs do not require p-eIF2α or translation elongation to release mRNAs.** *A*, IF for G3BP1 and PABPC1 in parental (WT) and eIF2α–S51A knockin MEFs transfected with or without poly(I·C). *B*, immunoblot analysis for RNase L in WT and eIF2α–S51A MEF cell lines transduced with RNase L–encoding lentivirus. *n.s.* indicates nonspecific band for the purpose of showing equal loading. *C*, similar to *A* but in MEF-RL and MEF–eIF2α–S51A-RL cell lines from *B. D*, IF for G3BP1 in WT and RL-KO cells either transfected with poly(I·C) or treated with 500 μm sodium arsenite (*SA*) and simultaneously mock-treated or treated with ISRIB or emetine. *E*, mean area of G3BP1+ foci in WT (RLB) or RL-KO (SG) from cells represented in *D. F*, Western blotting for GADD34 from parental and GADD34-KO A540-RL-KO cells six h following either poly(I·C) transfection or sodium arsenite treatment. *n.s.* indicates nonspecific band for the purpose of showing equal loading. *G*, time in which individual cells (*dots*) contain SGs in RL-KO and RL/G34-KO cells from Movies S8 and S9. *H*, analysis of the percent of cells that assemble, disassemble, and reassemble SGs in RL-KO and RL/G34-KO cells from Movies S8 and S9. *I*, puromycin labeling of newly synthesized proteins and IF for PABPC1 in RL-KO cells either transfected with poly(I·C) or treated with 500 μm sodium arsenite and co-treated with or without ISRIB.

WT MEFs generated SGs in response to poly(I·C) (Fig. 4*A*). This is consistent with MEFs expressing very little RNase L (25), because stable expression of RNase L in MEFs resulted in an increase in the fraction of cells generating RLBs instead of SGs (Fig. 4, *B* and *C*). Importantly, MEF–eIF2α–S51A cells did not assemble SGs in response to poly(I·C) (Fig. 4*A*), demonstrating that dsRNA-induced SGs require p-eIF2α for their formation. However, a small fraction of MEF–eIF2α–S51A cells (∼1%) generated RLBs (Fig. 4*A*). Moreover, stable expression of RNase L in MEF–eIF2α–S51A cells resulted in a substantial fraction of cells (∼50%) displaying RLB assembly and nuclear PABPC1 accumulation (Fig. 4, *B* and *C*), a hallmark of RNase L activation (18). Thus, unlike SGs, RLBs do not require p-eIF2α for their biogenesis in response to dsRNA.

To further confirm that RLBs do not require p-eIF2α for their assembly, we tested whether ISRIB, which bypasses p-eIF2α–mediated translational repression and blocks canonical SG assembly (26), inhibits RLB assembly. As expected, arsenite-induced SGs were inhibited by ISRIB (Fig. 4, *D* and *E*). In contrast, poly(I·C)-induced RLBs were not inhibited by ISRIB, consistent with their ability to form independently of eIF2α phosphorylation.

Unexpectedly, dsRNA-induced SGs in RL-KO cells were also unaffected by ISRIB (Fig. 4, *D* and *E*), consistent with findings from an independent study (17). This is despite their dependence on PKR and p-eIF2α (Figs. 1*B* and 2*A*), as well as the ability of GADD34 to promote their disassembly (Fig. 4, *F–H*, and Movies S8 and S9). These results contradict the current model whereby PKR-mediated translation shut-off is through p-eIF2α, and GADD34 is presumed to restore translation by promoting dephosphorylation of p-eIF2α.

Examination of translation by puromycin staining in ISRIB-treated, SG-positive RL-KO cells showed that these cells indeed maintained translational repression (Fig. 4*I*), whereas ISRIB reversed translation repression and SG assembly in response to arsenite stress as previously described (26). Because dsRNA-mediated translation repression in RL-KO cells requires PKR (18), this suggests that PKR-mediated phosphorylation of eIF2α is required but not sufficient for translational repression and SG assembly. Therefore, in addition to regulating p-eIF2α, PKR- and GADD34-mediated regulation of translation requires an additional unknown mechanism.

To determine whether dsRNA-induced SGs and RLBs require release of nontranslating mRNAs from ribosomes, we treated cells with emetine, which inhibits SG formation by locking mRNAs in elongating ribosomes (27). As expected, SA-induced SGs were inhibited by emetine treatment in both WT and RL-KO cells (Fig. 4, *D* and *E*). Emetine treatment inhibited dsRNA-induced SGs in RL-KO cells, whereas RLBs in WT cells were unaffected. These data indicate that dsRNA-induced SGs, but not RLBs, require continued translation elongation for release of mRNAs from ribosomes.

**Figure 5.**
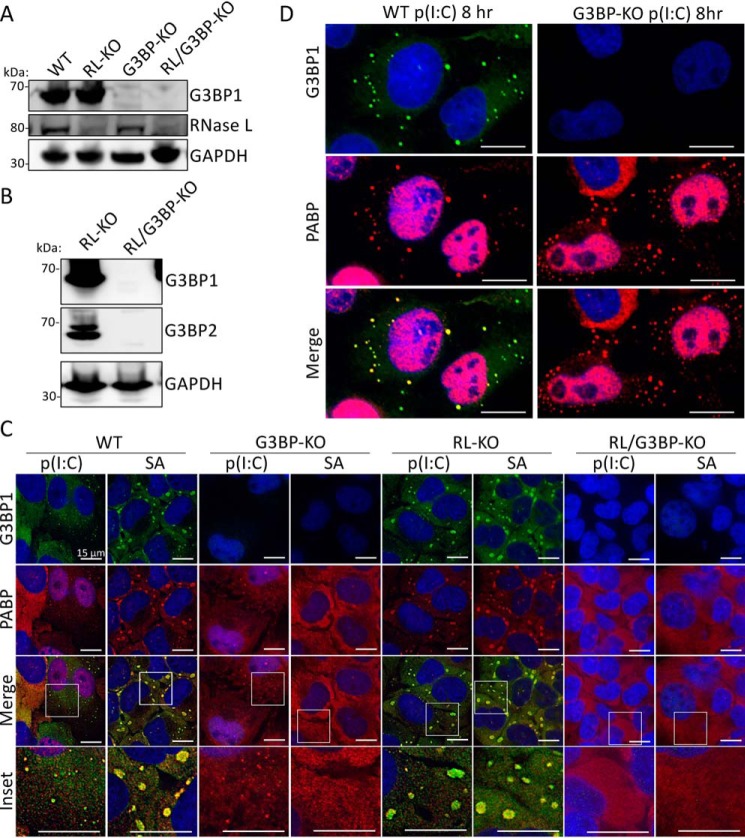
**dsRNA-induced SGs require G3BP1 and G3BP2, whereas RLBs do not.** *A*, immunoblot for G3BP1, RNase L, GAPDH in parental (WT), RL-KO, G3BP-KO, and RL/G3BP-KO U-2 OS cells. *B*, immunoblot for G3BP1 and G3BP2 in RL-KO and RL/G3BP-KO U-2 OS cells. *C*, IF for G3BP1 and PABPC1 4 h post-poly(I·C) transfection or treatment with 500 μm sodium arsenite (*SA*) in the indicated cell lines. *D*, similar to *C* but enlarged to show RLB formation in U-2 OS-G3BP1/2-KO cells.

Stress granules typically require the G3BP1 and G3BP2 paralogous RNA-binding proteins for their assembly (27). Therefore, we examined whether the formation of SGs and RLBs in response to dsRNA depends on G3BP for their biogenesis in WT and RL-KO U-2 OS cell lines (Fig. 5, *A* and *B*). As expected, both WT and RL-KO cells generated canonical SGs in response to sodium arsenite, whereas neither G3BP-KO nor RL/G3BP-KO cell lines generated SGs in response to SA treatment (Fig. 5*C*). Importantly, SGs did not form in RL/G3BP-KO cells, whereas RLBs still formed in the G3BP-KO cells in response to poly(I·C) (Fig. 5, *C* and *D*). Thus, unlike SGs, RLBs do not require G3BP for their assembly. Combined, these data indicate that RLBs have a distinct biogenesis pathway from dsRNA-induced SGs in that RLBs can form independently of p-eIF2α, release of mRNAs from ribosomes via translation elongation, and G3BP1/2.

### PABPC1 is dynamically associated with RLBs in contrast to SGs

**Figure 6.**
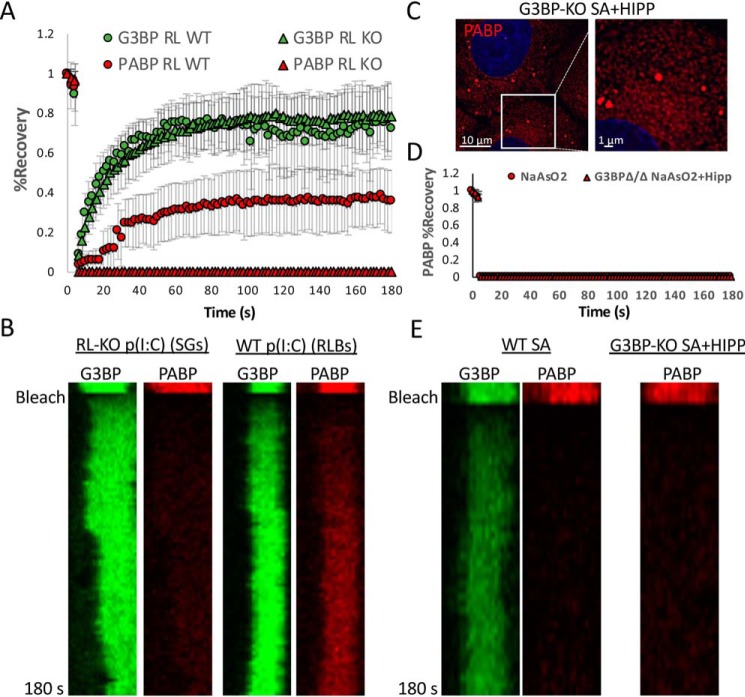
**PABP is more dynamically associated with RLBs than SGs.** *A*, FRAP of GFP–G3BP1 and mRuby-2–PABPC1 in WT and RL-KO A549 cells. *B*, kymograph of FRAP analysis represented in *A. C*, PABPC1–mRuby-2 fluorescence in U-2 OS-G3BP1/2-KO cells treated with sodium arsenite and hippuristanol. *D*, FRAP of mRuby-2–PABPC1 in U-2 OS-G3BP1/2-KO cells treated with sodium arsenite and hippuristanol. *E*, kymograph of FRAP analysis represented in *D*.

To determine whether the biophysical properties of RLBs are different from SGs, we examined protein dynamics of dsRNA-induced SGs and RLBs. For this experiment, we generated WT or RL-KO A549 cells that stably express mRuby-2–PABPC1 and eGFP–G3BP1 via lentiviral transduction. We then examined the dynamics of these proteins in dsRNA-induced SGs and RLBs by FRAP. G3BP1 readily recovered in dsRNA-induced SGs and in RLBs, demonstrating that the exchange rate of G3BP1 is similar between SGs and RLBs (Fig. 6, *A* and *B*). In contrast, PABPC1 did not recover in SGs but showed increased recovery in RLBs. The ability of PABP to recover in RLBs and not SGs is not due to the smaller size of RLBs, because hippuristanol/arsenite-induced SGs in G3BP-KO cells (28), which are similar in size to RLBs, do not recover PABP (Fig. 6, *C–E*). Thus, dsRNA-induced RLBs and SGs have distinct biophysical interactions of PABP that may underlie their biogenesis and maintenance. Moreover, the ability of PABP and G3BP1 to recover after photobleaching in RLBs further indicates that they have liquid-like properties.

## Discussion

We present several lines of evidence that a unique type of RNP granule distinct from SGs and P-bodies, referred to as an RLB, forms in response to dsRNA-mediated activation of RNase L. This was first suggested by the observation that dsRNA-induced G3BP1-positive foci are much smaller and more spherical than typical SGs in cells that have active RNase L (18). Additional evidence that RLBs are different from stress granules is that they form in a greater fraction of cells than SGs, have faster assembly kinetics than SGs in response to dsRNA, form independently of PKR and phosphorylation of eIF2α (Figs. 1, *A* and *B*, and 4, *A–C*), are unaffected by emetine treatment (Fig. 4*D*), contain poly(A)+ mRNA but not intact mRNAs (Figs. 1*F* and 3, *B–E*), exhibit different dynamics of PABP (Fig. 6, *A* and *B*), and contain only a subset of RBPs that localize to canonical SGs (Fig. 2).

Interestingly, a number of viral infections have been described to trigger the formation of small discrete “stress granules” instead of canonical SGs, based on the inclusion of G3BP, PABP, TIA1, and/or poly(A) in those assemblies (29–32). Because we now know G3BP, PABP, and poly(A) RNA are in RLBs and small TIA1 foci are observed during RNase L activation (Figs. 1 and 2), we suggest that many of those small viral specific stress granules will in fact be RLBs and/or are the result of RNase L activation. Although RLBs precede formation of SGs in A549 cells, we show that RLBs can also be generated from pre-existing SGs (Fig. 3). Therefore, the kinetic responses between PKR and RNase L activation will dictate whether cells display SGs or RLBs, and this could be specific to the cell type, virus, multiplicity of infection, and time after infection. An important issue to address in future work will be to examine the consequences of RNase L–mediated conversion of SGs to RLBs, the mechanism(s) that drive *de novo* RLB formation, and the biological significance of RLBs and SGs during viral infection.

An unresolved issue is the signal that triggers RLB formation. The observation that RLB bodies only form when RNase L is active suggests two possibilities. First, it could be that the widespread degradation of cytosolic RNA allows for the self-assembly of a number of RNA-binding proteins through interactions that are normally limited by RNA (33). This model would predict that RLBs would form in response to any widespread degradation of cytosolic RNA. However, cytoplasmic PABP-positive foci have not been reported during mRNA decay mediated by the KSHV SOX endoribonuclease (34–36). This suggests that RLBs are specific to RNase L–mediated RNA decay and may form because of nucleation of specific RNA fragments generated by RNase L via a combination of RNA–RNA and protein–protein interactions. Because RNase L can target ribosome-associated mRNAs (18), this could explain why RLBs do not require inhibition of translation initiation or continued elongation for ribosome runoff to release mRNAs for RLB assembly. Second, it could be that RNase L–mediated RNA degradation triggers a signaling pathway, other than phosphorylation of eIF2α, that promotes RLB formation. An important goal in future work will be to further understand the mechanism of RLB formation to manipulate their assembly and interrogate their function.

Our work reveals a fundamental difference between mRNAs in P-bodies and those in SGs or distributed in the cytosol. Specifically, we observe that P-bodies persist with no observable change when RNase L is activated or when translation is repressed by PKR in response to dsRNA. Because P-bodies are dependent on RNAs for their formation in yeast (37), this implies that either P-body–bound RNAs are resistant to RNase L or that mammalian P-bodies differ in their dependence on RNA for their maintenance during dsRNA stress. An important topic to address in future studies will be to determine why neither RNase L–mediated RNA decay nor PKR-mediated translational repression alter P-body assembly and whether this has a functional consequence, such as P-bodies storing and protecting specific mRNAs from RNase L–mediated decay.

Our live-cell imaging and FRAP analyses revealed that RLBs exhibit liquid-like properties, because they are spherical, have the ability to fuse, and are dynamic. Interestingly, RLBs closely and stably associate with P-bodies, which exhibit many similar liquid-like properties. However, we did not observe fusion between RLBs and P-bodies. Thus, RLBs and P-bodies represent an example of distinct RNP condensates that extensively interact. Because P-bodies are enriched in mRNA-decay machinery and RLBs form during RNase L–mediated RNA decay, it is tempting to speculate that RLBs could function in facilitating mRNA partitioning to P-bodies for degradation. Future work will focus on exploring the interactions between RLBs and P-bodies and assessing whether theses interactions play a role in the RNase L–mediated mRNA decay.

## Experimental procedures

### Plasmids

PKR-targeting sgRNAs were designed using IDT sgRNA design tool. PKR-targeting sgRNA oligonucleotides (IDT: PKR_sgRNA_1_sen, CACCGATTCAGGACCTCCACATGAT; PKR_sgRNA_1_anti, CATCATGTGGAGGTCCTGAATCAAA; PKR_sgRNA_2_sen, CACCGTTATCCATGGGGAATTACAT; and PKR_sgRNA_2_anti, CATGTAATTCCCCATGGATAACAAA) were ligated into the BbsI sites I px458-GFP-Cas9 plasmid (Addgene catalog no. 48138) using T4 ligase (NEB). The G3BP1-, G3BP2-, and GADD34-targeting Cas9 vectors were generated similarly using the following oligonucleotides: G3BP1_sgRNA_1_sen, CACCGTACCACACCATCATTTAGCG; G3BP1_sgRNA_anti, AAACCGCTAAATGATGGTGTGGTA; G3BP2_sgRNA_sen, CACCGGAGTGATGGAGTAGTTGTCC; G3BP2_sgRNA_anti, AAACGGACAACTACTCCATCACTCC; GADD34_sgRNA1_sen, CACCGGGACAACACTCCCGGTGTGA; GADD34_sgRNA1_anti, AAACTCACACCGGGAGTGTTGTCCC; GADD34_sgRNA_2_sen, CACCGTGAACGATACTCCCAGGACC; and GADD34_sgRNA_2_anti, AAACGGTCCTGGGAGTATCGTTCAC. The pLJM1–eGFP–G3BP1 vector was made by subcloning the eGFP–G3BP1 coding sequence into the NheI/EcoRI sites in pLJM1–eGFP. To make the pLenti–eGFP–G3BP1 vector, the eGFP–G3BP1 coding sequence was amplified via PCR using Phusion polymerase and inserted into the XhoI/XbaI sites of pLenti–EF1–BLAST vector using in-fusion. To generate the pLenti–mRuby-2–PABPC1 lentiviral plasmid, the mRuby-2 coding sequence was amplified via PCR, the PABPC1 coding sequence was amplified via PCR from the pCI–MS2V5–PABPC1 (Addgene catalog no. 65807), and the sequences were fused with and inserted into the XhoI/XbaI sites of pLenti–EF1–BLAST vector using in-fusion.

### Antibodies

DCP1B (D2P9W) Rabbit mAb (Cell Signaling Technology catalog no. 13233) was used at 1:500 for IFA. Mouse anti-RNase L antibody 2E9 (Novus Biologicals catalog no. NB100-351) was used at 1:1500 for immunoblot analyses. Mouse monoclonal anti-G3BP antibody (Abcam catalog no. ab56574) was used at 1:1000 for IFA and for IB analyses. Rabbit anti-GAPDH (Cell Signaling Technology catalog no. 2118L) was used at 1:2000 for IB analysis. Rabbit anti-PKR (Cell Signaling Technology catalog no. 12297S) was used at 1:1000 for IB analysis. Rabbit polyclonal anti-PABP antibody (Abcam catalog no. ab21060) was used at 1:1000 for IFA. Rabbit polyclonal anti-TIA1 (Abcam catalog no. ab40693) was used at 1:500 for IFA. Rabbit anti-FAM120A (Sigma–Aldrich catalog no. HPA019734) was used at 1:500 for IFA. Rabbit polyclonal anti-Caprin1 (Fisher Scientific catalog no. 50–554-357) was used at 1:500 for IFA. Rabbit polyclonal anti-PUM1 (Thermo Fisher Scientific catalog no. PA5-30327) was used at 1:500 for IFA. Rabbit polyclonal anti-FMRP (Abcam catalog no. ab17722) was used at 1:500 for IFA. Anti-GFP (Invitrogen catalog no. A11122) and IgG (Invitrogen catalog no. 10500C) were used for RLB immunoprecipitation. Goat anti-mouse IgG FITC (Abcam catalog no. ab97022) was used at 1:1000 for IFA. Goat anti-rabbit IgG Alexa Fluor 647 (Abcam catalog no. ab150079) was used at 1:1000 for IFA. Anti-rabbit IgG, HRP-linked antibody (Cell Signaling Technology catalog no. 7074S) was used at 1:3000 for IB analysis. Anti-mouse IgG, HRP-linked antibody (Cell Signaling Technology catalog no. 7076S) was used at 1:10,000 for IB analysis. Anti-puromycin antibody was used at 1:1000 for IFA (Millipore–Sigma catalog no. MABE343).

### Cell culture, drug treatments, and transfections

The A549 cell line was provided by Dr. Chris Sullivan (38). The U-2 OS, U-2 OS G3BP1/2-KO, and U-2 OS GFP–G3BP/mRFP–DCP1a cells were provided by Dr. Paul Anderson (27, 39). The cells were maintained at 5% CO_2_ and 37 °C in Dulbecco's modified Eagle's medium supplemented with fetal bovine serum (10%, v/v) and penicillin/streptomycin (1% v/v). The cells were routinely tested for mycoplasma contamination by the CU Boulder BioFrontiers cell culture core facility and were negative for mycoplasma contamination throughout the study. The cells were transfected with high-molecular-weight poly(I·C) (InvivoGen catalog no. tlrl-pic) using 3 μl of Lipofectamine 2000 (Thermo Fisher Scientific) per 1 μg of poly(I·C). Unless otherwise noted, 500 ng/ml of poly(I·C) was used. Low-molecular-weight poly(I·C) (InvivoGen catalog no. tlrl-pic) was also used where indicated. Pateamine A was provided by Dr. Jerry Pelletier (Department of Biochemistry, McGill University), and the cells were treated with 100 nm pateamine A. The cells were treated with 500 μm of sodium arsenite (Sigma–Aldrich).

### Generation of knockout cell lines

Generation of knockout cell lines was performed as described in Ref. 18. Briefly, to knock out PKR in A549 and A549-RL-KO cell lines, cells (T-25 flask; 70% confluent) were co-transfected with 2 μg of px458-PKR and 200 ng of pcDNA3.1-puro using 6 μl of Lipofectamine 2000 (Thermo Fisher Scientific) according to the manufacturer's instructions. Twenty-four hours post-transfection, the medium was replaced with medium containing 2 μg/ml of puromycin. Selective medium was replaced 3 days post-transfection. Five days post-transfection, selective growth medium was replaced with normal growth medium. The cells were serial diluted and plated on 15-cm dishes. Individual colonies were isolated, propagated, and screened via Western blotting analysis. Knockout of GADD34 in A549-RL-KO cells was done similarly.

### Generation of lentiviral particles and stable cell lines

Generation of lentivirus was performed as described in Ref. 18. Briefly, to generate the GFP–G3BP1 and mRuby-2–PABPC1 lentiviral particles, HEK293T cells (T-25 flask; 80% confluent) were co-transfected with either 2.7 μg of either pLenti–EF1–GFP–G3BP1–blast or pLenti–EF1–mRuby-2–PABPC1–blast, 870 ng of pVSV-G, 725 ng of pRSV–Rev, and 1.4 μg of pMDLg–pRRE using 20 μl of Lipofectamine 2000. Medium was replaced 6 h post-transfection. Medium was collected at 24 and 48 h post-transfection and filter-sterilized with a 0.45-μm filter. To generate the A549 WT and RL-KO GFP–G3BP1/mRuby-2–PABPC1 stable cell lines, WT or RL-KO A549 cells (T-25 flask; 80% confluent) were transduced with 1 ml of lentiviral stocks containing 10 μg/ml of Polybrene for 1 h. Normal medium was then added to the flask. 24 h post-transduction, the cells were reseeded in T-25 flask containing 5 μg/ml of blasticidin selective medium. The cells were maintained in selective medium for 4 days before returning to normal growth medium.

To generate GFP–G3BP1 lentiviral particles for transducing WT and RL-KO U-2 OS cells, HEK293T cells (15-cm dish; 80% confluent) were co-transfected with 11.7-μg of pLMJ1–GFP–G3BP1, 3.5 μg of pVSV-G, 2.9 μg of pRSV–Rev, and 5.6 μg of pMDLg–pRRE using 100 μl of Lipofectamine 2000. The medium was collected at 24 and 48 h post-transfection and filter-sterilized with a 0.45-μm filter. WT and RL-KO U-2 OS cells (T-25 flask, 80% confluent) were incubated with 1 ml of GFP–G3BP1 lentivirus particles (6.4 × 10^5^ IU/ml; multiplicity of infection of ∼0.5) containing 10 μg/ml of Polybrene for 1 h. Normal medium was then added to the flask. 24 h post-transduction, the cells were reseeded in T-25 flask containing 2-μg/ml Puromycin selective medium. The cells were maintained in selective medium for 4 days before returning to normal growth medium.

### Western blotting analysis

Western blotting analysis was performed as described in Ref. 18. The cells were lysed in SDS solution (1% SDS, 2% β-mercaptoethanol) by boiling for 10 min followed by 1 min of vortexing. Equal volumes of lysates were fractionated on 4–12% Bis-Tris protein gels (Thermo Fisher Scientific) in MES buffer and transferred to nitrocellulose membrane (GE Healthcare). The membranes were blocked in 5% BSA in TBST. The membranes were then incubated with primary antibodies overnight at 4 °C. After washing, the membranes were incubated with HRP-linked anti-rabbit IgG or anti-mouse IgG secondary antibodies for 1 h at room temperature. After washing, the membranes were incubated with ECL substrates (Thermo Fisher Scientific catalog no. 32106) for 1–5 min. The membranes were then stripped using Restore Western blotting stripping buffer (Thermo Fisher Scientific catalog no. 21059) and reblocked with 5% BSA in TBST. Photographs of membranes were taken using ImageQuant LAS 4000 (GE Healthcare) and analyzed using ImageJ with Fiji plug-in.

### Microscopy

Immunofluorescence and smFISH with 4′,6′-diamino-2-phenylindole staining were imaged using a wide field DeltaVision Elite microscope with a 100× objective using a PCO Edge sCMOS camera. For IFA, 10 Z sections at 0.3 μm/section were taken for each image. For IFA/smFISH, 15 Z planes at 0.2 μm/section were taken for each image. Deconvoluted images were processed using ImageJ with FIJI plugin. Z-planes were stacked, and minimum and maximum display values were set in ImageJ for each channel to properly view fluorescence. Quantification of smFISH was determined using Imaris Image Analysis Software (Bitplane) (University of Colorado–Boulder, BioFrontiers Advanced Light Microscopy Core). Live-cell imaging was performed using a Nikon spinning-disk confocal microscope outfitted with an environmental chamber with O_2_, CO_2_, temperature, and humidity control (University of Colorado–Boulder, BioFrontiers Advanced Light Microscopy Core). All images were acquired using a 2× Andor Ultra 888 EMCCD camera.

FRAP assays were performed using an inverted Nikon A1R laser scanning confocal microscope equipped with an environmental chamber, a 100× NA 1.5 oil objective, and Nikon Elements software. Cells expressing GFP–G3BP and mCherry–PABP were transfected with poly(I·C). 3 h later, the cells were then placed in the Nikon A1R environmental chamber at 37 °C, 5% CO_2_. SG regions of varying sizes were selected for photobleaching. FRAP was performed by bleaching selected areas with 100% laser power for GFP and mCherry channels and then subsequently monitoring recovery of GFP and mCherry simultaneously over a period of 3 min. Three SGs were selected per cell with five cells/condition. To analyze recovery, the mean intensity of each bleached region was quantified in ImageJ, and recovery intensities were normalized to the mean prebleach measurements. Mobile fractions ϕM were computed by subtracting the minimum normalized mean intensity *I*_0_ from the normalized end-point intensity IF: ϕM = IF − I0. To determine *t*_½_, the data were fit in to the equation: *f*(*t*) = *A*(1 − *e* (−τ;*t*)) + *c*, where *f*(*t*) = % recovery, *A* = mobile fraction, *t* = time, and τ = *t*_½_. Using % recovery, time, and calculated mobile fractions (*A*) as an upper constraint, *t*_½_ was determined using Excel Solver.

### Mass spectrometry

U-2 OS-GFP–G3BP1 cells were grown to 80% confluence in 15-cm dishes (two dishes/replicate). The cells were transfected with poly(I·C) at 0.5 μg/ml. 4 h post-transfection, the medium was then aspirated, and the cells were resuspended in medium, scraped into a 50-ml conical tube, and pelleted via centrifuged at 1500 × *g*. The supernatant was aspirated, and the pellets were snap-frozen in liquid nitrogen. After thawing, the cells were resuspended in 1 ml of stress granule lysis buffer (50 mm Tris-HCl, pH 7.4, 100 mm potassium acetate, 2 mm magnesium acetate, 0.5 mm DTT, 50 μg/ml heparin, 0.5% Nonidet P-40, 1 complete mini EDTA free protease inhibitor tablet per 50 ml of buffer). The cells were then passed through a 25-gauge 5/8 needle seven times on ice to lyse. At this step, the lysate was inspected by wide field microscopy to determine whether granules were visible in the medium. The cells were then pelleted by centrifugation at 300 × *g* for 5 min at 4 °C. The supernatant was taken RNP complexes were pelleted via centrifugation at 18,000 × *g* for 20 min at 4 °C. The pellet was resuspended in 1 ml of stress granule lysis buffer and pelleted via centrifugation at 18,000 × *g* for 20 min at 4 °C. To preclear the samples and remove nonspecific binders, the pellet was resuspended in 340 μl of lysis buffer, and 60 μl of prewashed protein A Dynabeads were added and incubated for 30 min at 4 °C on nutator. Dynabeads were then taken off twice using a magnet, and the preclearance step was repeated. Following final removal of beads, 1 μg of either anti-GFP antibody (Invitrogen catalog no. A11122) or anti-IgG (Invitrogen catalog no. 10500C) were added to the respective samples and incubated overnight on nutator at 4 °C.

Following incubation, the samples were centrifuged at 18,000 × *g* for 20 min at 4 °C to remove antibody. The pellet was resuspended in 500 μl of stress granule lysis buffer, and 33 μl of washed protein A Dynabeads (1 mg) were added and nutated for 3 h at 4 °C. The beads were then washed for 2 min in wash buffer 1 (stress granule lysis buffer + 2 m urea), for 5 min in wash buffer 2 at 4 °C (stress granule lysis buffer + 300 mm of potassium acetate), and for 5 min with stress granule lysis buffer at 4 °C. The sample was then washed eight times with 1 ml of tris-EDTA (TE) buffer to remove detergent, and the beads were brought up in 50 μl of TE buffer.

The samples were then processed by the MS facility at University of Colorado–Boulder and analyzed on Thermo LTQ Orbitrap (Thermo Fisher Scientific). The Andromeda search engine was used to map peptides against the Uniprot human protein sequence database (71,803 entries) downloaded on January 12, 2018 (40). Parameters for mapping are included in Data File S1. Only identification, reverse, or potential contaminants were filtered out. The false discovery rate was calculated as described in Refs. 40 and 41. Proteins with fewer than five cumulative spectral counts between the three replicates were removed. Analysis of the variance between the GPF–G3BP1 immunoprecipitated replicates showed that the proteins enriched are reproducible (Rep1 and Rep2: *R*^2^ = 0.774, Rep2 and Rep3: *R*^2^ = 0.929, Rep1 and Rep3: *R*^2^ = 0.721). The spectral counts from the remaining proteins were averaged and divided by the spectral counts in the IgG control. Proteins that were 2-fold enriched over the IgG control were selected for further analysis. A stress granule reference file was created by merging the proteins identified in three different stress granule proteomic studies that stress cells with sodium arsenite (3, 42, 43), which resulted in a stress granule proteome of 491 proteins. To determine the overlap between the poly I:C granule proteome and the sodium arsenite stress granule proteome, the two protein lists were inner joined using *R*. Gene Ontology was performed on the proteins that did not overlap with the stress granule proteome. Gene ontology biological processes were derived from Gene Ontology Consortium enrichment analysis (44).

### Data availability

Raw mass spectrometry data sets were deposited in Mendeley: Burke, J. (2019), RNase L promotes the formation of unique ribonucleoprotein granules distinct from stress granules, Mendeley Data, V1, doi: 10.17632/gy3br29tzr.1.

## Author contributions

J. M. B. and R. P. conceptualization; J. M. B., E. T. L., and D. T. data curation; J. M. B., E. T. L., D. T., and R. P. formal analysis; J. M. B. and R. P. funding acquisition; J. M. B., E. T. L., and D. T. investigation; J. M. B. and R. P. writing-original draft.

## Supplementary Material

Supporting Information

## Footnotes

2 The abbreviations used are:

SG: stress granule
RL and RNase L: ribonuclease L
PKR: protein kinase R
RLB: RNase l-dependent body
poly(I·C): polyinosinic:polycytidylic acid
RNP: ribonucleoprotein
eIF: eukaryotic translation initiation factor
IF: immunofluorescence
MEF: mouse embryonic fibroblast; immunoblotting
HRP: horseradish peroxidase
FRAP: fluorescence recovery after photobleaching
IFA: immunofluorescence assay
MS: mass spectrometry.
