# Supplementary material for: RNase L promotes the formation of unique ribonucleoprotein granules distinct from stress granules
Source: J Biol Chem. 2020 Jan 2;295(6):1426–38. doi: 10.1074/jbc.RA119.011638 (PMC7008361; doi:10.1074/jbc.RA119.011638)
Supplement: Supporting Information [file supp_295_6_1426__index.html]

RNase L promotes the formation of unique ribonucleoprotein granules distinct from stress granules — RNase L regulates RNP granule assembly — RNase L promotes the formation of unique ribonucleoprotein granules distinct from stress granules — EDITORS' PICK: RNase L regulates RNP granule assembly — Supporting Information 

# RNase L promotes the formation of unique ribonucleoprotein granules distinct from stress granules

## Supporting Information

- Movie 1. RLBs and P-bodies extensively and stably interact. - U2 OS cells stably expressing GFP-G3BP1 (RLB marker) and RFP-DCP1a (P-body marker) were transfected with poly(I:C). Images were captured between one- and two-hours post-poly(I:C) every ten seconds. The videos speed is five frames per second.
- Movie 2. RLBs and SGs transiently interact. - U2 OS cells stably expressing GFP-G3BP1 (RLB marker) and RFP-DCP1a (P-body marker) were treated with 100nM pateamine A. Images were captured between one- and two-hours post-treatment every thirty seconds. The videos speed is five frames per second.
- Movie 3. RLBs and P-bodies undergo homotypic fusion, but do not fuse with one another. - An inset from Movie 1 to show homotypic fusion events between P-bodies (Red) and RLBs (green).
- Movie 4. RNase L disassembles SGs - U-2 OS cells expressing GFP-G3BP1 were treated with pateamine A (100nM) for 1 hour. Cell were then transfected with poly(I:C). Time indicates minutes post-poly (I:C).
- Movie 5. Poly(I:C) transfection does not affect pateamine A-induced SGs in RL-KO cells - U-2 OS-RL-KO cells expressing GFP-G3BP1 were treated with pateamine A (100nM) for 1 hour. Cells were then transfected with poly(I:C). Time indicates minutes post-poly (I:C).
- Movie 6. Biogenesis and maintenance of RLBs - A549 cells expressing mRuby2-PABPC1 were transfected with poly(I:C). Time indicates minutes post-poly (I:C).
- Movie 7. RLBs form independently of SGs - A549 cells expressing GFP-G3BP1 and mRuby2-PABPC1 were transfected with poly(I:C). Images were captured between one- and two-hours following transfection.
- Movie 8. Biogenesis and maintenance of SGs in RL-KO cells - A549-RL-KO cells expressing mRuby2-PABPC1 were transfected with poly(I:C). Time indicates minutes post-poly (I:C).
- Movie 9. Biogenesis and maintenance of SGs in RL/GADD34-KO cells - A549-RL/GADD34-KO cells expressing mRuby2-PABPC1 were transfected with poly(I:C). Time indicates minutes post-poly (I:C).
- Data File 1. Mass spectrometry analyses of RLBs - Raw and processed mass spectrometry data of RLB-associated proteins.
